# Polyglactin 910 Meshes Coated with Sustained-Release Cannabigerol Varnish Inhibit *Staphylococcus aureus* Biofilm Formation and Macrophage Cytokine Secretion: An In Vitro Study

**DOI:** 10.3390/ph16050745

**Published:** 2023-05-13

**Authors:** Mustafa Abudalu, Muna Aqawi, Ronit Vogt Sionov, Michael Friedman, Irith Gati, Yaron Munz, Gil Ohana, Doron Steinberg

**Affiliations:** 1 The Biofilm Research Laboratory, The Institute of Biomedical and Oral Research (IBOR), The Faculty of Dental Medicine, The Hebrew University of Jerusalem, Jerusalem 9112102, Israel; muna.aqawi@mail.huji.ac.il (M.A.); dorons@ekmd.huji.ac.il (D.S.); 2 Department of General Surgery, Barzilai Medical Center, Ashkelon 7830604, Israel; munzy@bmc.gov.il (Y.M.); gilo@bmc.gov.il (G.O.); 3 The Institute of Drug Research, School of Pharmacy, The Hebrew University of Jerusalem, Jerusalem 9112102, Israel; michaelf@ekmd.huji.ac.il (M.F.); irith.gati@mail.huji.ac.il (I.G.)

**Keywords:** sustained-release varnish, *Staphylococcus aureus*, cannabigerol, biofilm, mesh, macrophages, cytokines

## Abstract

Synthetic surgical meshes are commonly used in abdominal wall reconstruction surgeries to strengthen a weak abdominal wall. Common mesh-related complications include local infection and inflammatory processes. Because cannabigerol (CBG) has both antibacterial and anti-inflammatory properties, we proposed that coating VICRYL (polyglactin 910) mesh with a sustained-release varnish (SRV) containing CBG would prevent these complications. We used an in vitro infection model with *Staphylococcus aureus* and an in vitro inflammation model of lipopolysaccharide (LPS)-stimulated macrophages. Meshes coated with either SRV-placebo or SRV-CBG were exposed daily to *S. aureus* in tryptic soy medium (TSB) or macrophage Dulbecco’s modified eagle medium (DMEM). Bacterial growth and biofilm formation in the environment and on the meshes were assessed by changes in optical density, bacterial ATP content, metabolic activity, crystal violet staining, spinning disk confocal microscopy (SDCM), and high-resolution scanning electron microscopy (HR-SEM). The anti-inflammatory effect of the culture medium that was exposed daily to the coated meshes was analyzed by measuring the release of the cytokines IL-6 and IL-10 from LPS-stimulated RAW 264.7 macrophages with appropriate ELISA kits. Additionally, a cytotoxicity assay was performed on Vero epithelial cell lines. We observed that compared with SRV-placebo, the segments coated with SRV-CBG inhibited the bacterial growth of *S. aureus* in the mesh environment for 9 days by 86 ± 4% and prevented biofilm formation and metabolic activity in the surroundings for 9 days, with respective 70 ± 2% and 95 ± 0.2% reductions. The culture medium that was incubated with the SRV-CBG-coated mesh inhibited LPS-induced secretion of IL-6 and IL-10 from the RAW 264.7 macrophages for up to 6 days without affecting macrophage viability. A partial anti-inflammatory effect was also observed with SRV-placebo. The conditioned culture medium was not toxic to Vero epithelial cells, which had an IC_50_ of 25 µg/mL for CBG. In conclusion, our data indicate a potential role of coating VICRYL mesh with SRV-CBG in preventing infection and inflammation in the initial period after surgery.

## 1. Introduction

Surgeons utilize synthetic mesh in abdominal wall reconstruction surgeries to achieve a more durable repair compared with traditional tissue repair [1]. Meshes allow the reinforcement of native tissue, resulting in tissue ingrowth and the lateralization of force across the abdominal wall [2]. Despite the many advantages of mesh implants in surgery, there are still many drawbacks. The most common complications associated with mesh implants are acute or delayed infections, adhesion, and inflammatory processes, which have become concerning clinical issues [3,4]. Mesh infections are associated with high morbidity, hospital readmissions, repeat surgeries, and impaired quality of life [5]. In addition, the economic impact of mesh infections is significant [2].

There are several types of meshes, which can be categorized into two major classes: synthetic and biological meshes. Synthetic meshes are either nondegradable or degradable, while biological meshes are all degradable [6]. Conventional nondegradable meshes are usually the least expensive with low recurrence rates. However, they are not recommended for infected areas and are associated with higher infection rates, discomfort, and adhesions [6]. To address these drawbacks, synthetic degradable meshes (e.g., Vicryl (Ethicon, Raritan, NJ, USA) and Dexon (American Cyanamid Co., Bridgewater Township, NJ, USA) were designed. They were intended to be placed in infected areas but have been associated with high recurrence rates as they degrade within one to three months [7,8]. Biological meshes have been expected to promote regeneration and can be used in infected/complex areas. However, they are very expensive and have a high recurrence rate [9]. The VICRYL polyglactin 910 woven mesh has the advantage of having great tensile strength with high flexibility, thus allowing excellent tissue support. The Vicryl suture also allows soft passage through the tissues with minimum sawing, tissue drag, and trauma. Another advantage is that it is absorbable and well tolerated.

Currently, nonsurgical treatment options for mesh infections are limited and include the systemic administration of antibiotics. Frequently, the infection of mesh necessitates its removal [10,11]. It has been reported that almost 70% of mesh explantations are associated with infection [1]. In general, contamination is thought to occur at the time of surgical insertion of the prosthesis into the abdominal cavity due to adherent microorganisms [12]. The most common microorganisms associated with mesh infection are *Staphylococcus aureus* and *Staphylococcus epidermidis*, which account for 90% of cases. However, infections with *Candida albicans*, *Pseudomonas aeruginosa*, *Escherichia coli*, *Streptococcus pyogenes*, *Klebsiella pneumoniae*, and *Enterococcus faecalis* have also been reported [13].

Biofilm formation is a major cause of staphylococcal infection consolidation [14]. A biofilm is an architectural community of microorganisms attached to a surface and embedded in a self-produced 3D matrix of extracellular polymeric substances [15,16]. The National Institutes of Health (NIH) documented that among all microbial and chronic infections, 65% and 80%, respectively, are associated with biofilm formation [17]. The biofilm provides a survival advantage to the microorganisms, among others, by reducing their sensitivity to antimicrobial agents and external hazardous stress stimuli [18,19,20].

Because surgical meshes are composed of synthetic materials, they induce immune responses involving the activation and recruitment of inflammatory cells to the site of implantation [21]. Previous studies demonstrated that the implantation of polyglactin 910 mesh attracts macrophages that infiltrate the mesh pores and the interstices between the braided filaments [22]. Macrophages are among the earliest immune cells recruited to sites of surgical tissue damage and play a critical role in the host response [23]. This initial inflammatory response can last several days, followed by a macrophage-centered response to stabilize the tissue and guide the healing process [24].

The topical use of sustained-release delivery systems offers several advantages over conventional delivery systems: they allow relatively high concentrations of drugs to be delivered locally, which facilitates drug diffusion into the lower layers of the biofilm, reduces side effects, and requires smaller amounts of the drug, thereby improving the therapeutic potential while increasing the safety [25,26]. Sustained-release varnishes (SRVs), which are based on polymers with active ingredients dissolved in solvents, are an example of these delivery systems. Varnishes are liquid pharmaceutical preparations that form a thin film when applied to a surface after drying [26]. SRVs enriched with various active compounds have been shown to provide long-term antimicrobial and antibiofilm protection in other medical systems, such as oral care [26], sinonasal cavity [27], and catheter-associated urinary tract infections [28].

There is growing interest in using the non-psychotropic compound cannabigerol (CBG) from the *Cannabis sativa* L. plant as an antibacterial [29,30,31] and anti-inflammatory agent [32,33,34]. The anti-inflammatory effects of CBG are, among others, mediated by its binding to CB2 cannabinoid receptors on immune cells [32,33,34] and by inhibiting cyclooxygenase-2 (COX-2) involved in prostaglandin synthesis [35]. Borrelli et al. [36] observed that CBG could prevent experimental inflammatory bowel disease in mice by reducing myeloperoxidase activity and inducible nitric oxide synthase (iNOS) expression, and it normalized the levels of the cytokines interleukin (IL)-1β, IL-10, and interferon-γ. CBG also has neuroprotective activities, acts as an antioxidant, and exerts antitumor effects [37,38,39]. CBG has been repeatedly shown to have antimicrobial effects with particularly great activity against Gram-positive bacteria, including methicillin-sensitive *Staphylococcus aureus* (MSSA), methicillin-resistant *Staphylococcus aureus* (MRSA), and *Streptococcus* spp., but also against the Gram-negative *Neisseria gonorrhoeae* and the fungus *Candida albicans* [29,30,40,41]. Appendino et al. [41] showed that CBG has antibacterial activity against several clinical MRSA isolates (e.g., SA-1199B, XU212, RN4220, EMRSA-15, and EMRSA-16). Likewise, Farha et al. [29] demonstrated the antibacterial activity of CBG against 96 clinical MRSA isolates with MIC values ranging from 2 to 8 μg/mL. Importantly, Farha et al. [29] repeatedly exposed MRSA to CBG and observed that resistance to CBG did not develop. CBG was also efficient against MRSA in an in vivo mouse model [29]. These observations make CBG a good candidate for an antibacterial drug that can be used in infections caused by both methicillin-sensitive and methicillin-resistant *S. aureus* strains. The aim of our study is to develop a sustained release technique that can be applied on biodegradable polyglactin 910 mesh to prevent bacterial infections, especially in the first critical phase after surgery.

Here, we propose a method to both prevent bacterial biofilm formation on surgical meshes and suppress inflammatory processes by coating a biodegradable VICRYL mesh (polyglactin 910) with cannabigerol (CBG)-containing SRV. We choose to use the VICRYL mesh because of its feasibility and low cost. CBG is chosen as the active ingredient because it is a non-psychoactive cannabinoid that has both antibacterial and anti-inflammatory properties [37]. *S. aureus* is chosen for the infection model because it is the most common pathogen causing mesh-related infections [13]. The in vitro inflammation model is performed with macrophages, which are innate immune cells involved in inflammatory processes associated with VICRYL meshes [23]. Here, we present data showing the antibacterial and anti-inflammatory effects of a VICRYL mesh coated with SRV-CBG using in vitro model systems. Our data indicate that our technology offers long-term drug release and prevents mesh-related bacterial infections and inflammation around the meshes, making it a potential strategy to improve surgical outcomes.

## 2. Results

### 2.1. VICRYL Meshes Coated with SRV-CBG and SRV-Placebo

**Figure 1:**
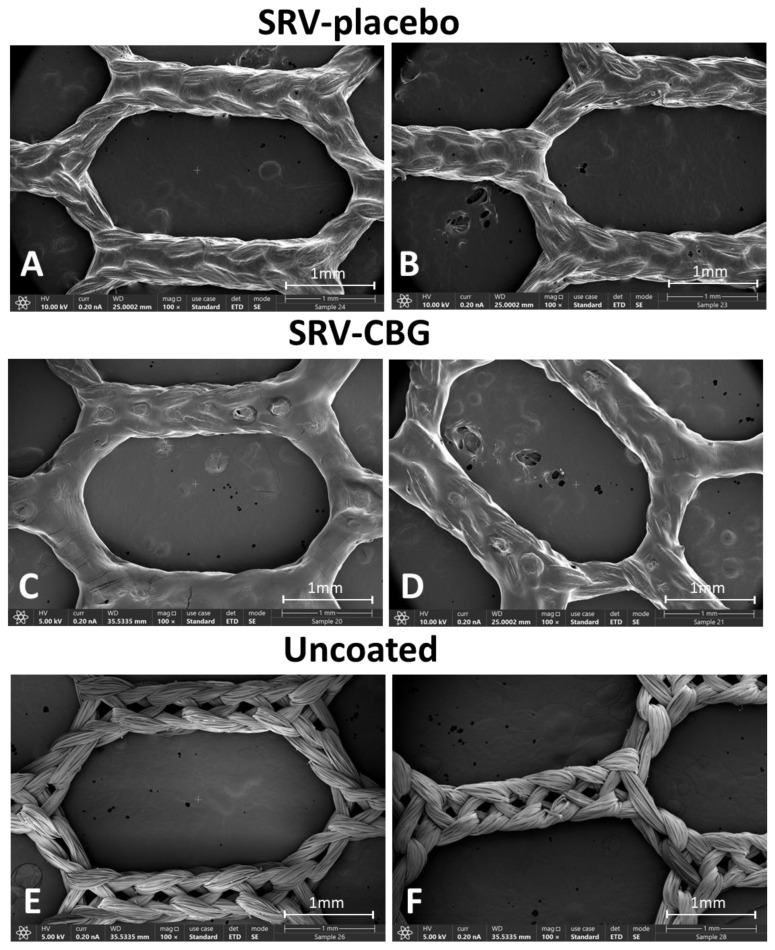
HR-SEM images of SRV-placebo-coated meshes at ×100 (**A**,**B**) magnification. HR-SEM images of SRV-CBG-coated meshes at ×100 (**C**,**D**) magnification. HR-SEM images of uncoated meshes at ×100 (**E**,**F**) magnification.

We first wanted to make sure that the varnish forms a good film on the polyglactin 910 mesh. HR-SEM images of the SRV-placebo (Figure 1A,B) and SRV-CBG (Figure 1C,D)-coated meshes show that the varnishes firmly formed films on the mesh fibers, leaving the macropores of the mesh open. The films formed by the placebo and CBG varnishes show some differences in thickness and roughness (Figure 1). HR-SEM images of uncoated mesh are shown in Figure 1E,F.

### 2.2. Antibacterial Effects of the SRV-CBG-Coated VICRYL Mesh on Staphylococcus aureus

CBG is effective against both methicillin-sensitive and methicillin-resistant *S. aureus* to a similar extent (Table 1), which is consistent with the observations of Appendino et al. [41] and Farha et al. [29]. Even a multidrug-resistant *S. aureus* (MDRSA) clinical isolate shows a similar sensitivity to CBG as the MSSA (Table 1), indicating the antibacterial action of CBG is not influenced by antibiotic-resistant mechanisms. Additionally, similar concentrations of CBG prevent biofilm formation of methicillin-sensitive and methicillin-resistant *S. aureus* (Table 1). Because there was no significant difference in the susceptibility of the different *S. aureus* strains to CBG, the *S. aureus* ATCC 25923 strain was selected for testing the antibacterial and antibiofilm activity of SRV-CBG-coated meshes.

**Figure 2:**
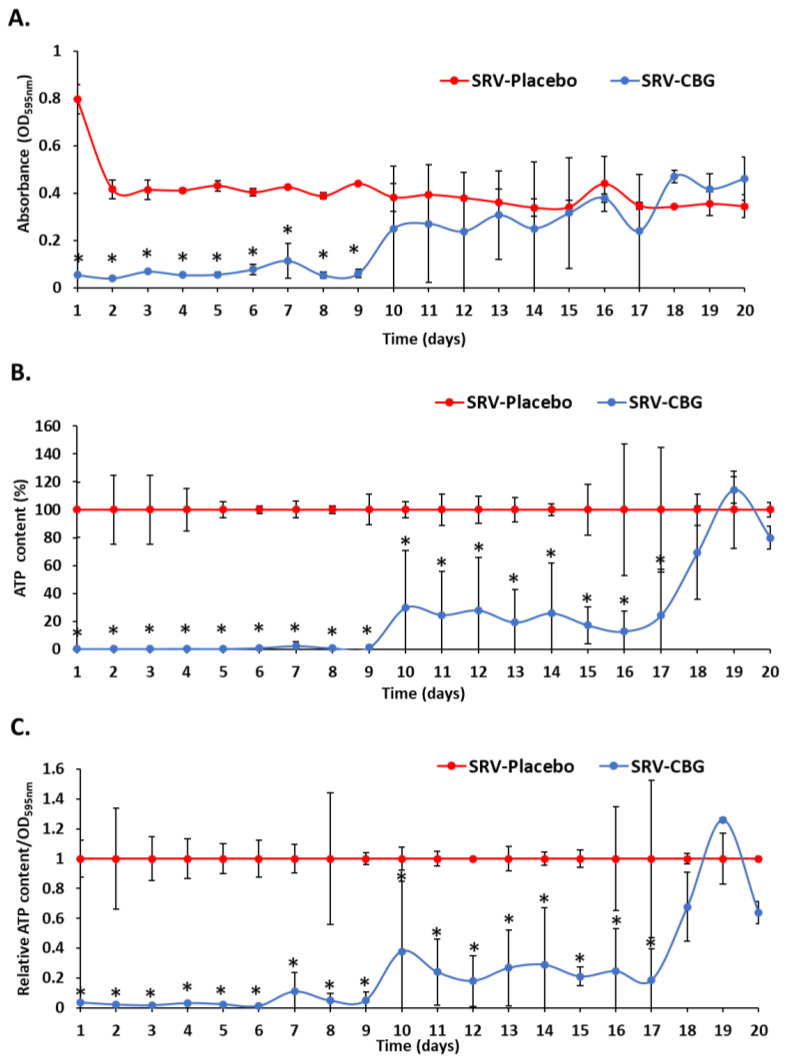
Antibacterial effects of the SRV-CBG-coated mesh on the planktonic growth of *S. aureus*. (**A**). SRV-CBG- and SRV-placebo-coated meshes were incubated daily with fresh *S. aureus* cultures for 20 days, and the OD at 595 nm of the supernatant was measured daily after a 24 h incubation period. *n* = 3. * *p* < 0.05 for SRV-CBG versus SRV-placebo, according to Student’s *t*-test. (**B**). The percentage of ATP content in *S. aureus* planktonic bacteria following 24 h exposure to SRV-CBG-coated meshes in comparison to ATP contents in *S. aureus* planktonic bacteria exposed to SRV-placebo-coated meshes. The kinetic study was conducted for 20 days. *n* = 3. * *p* < 0.05 for SRV-CBG versus SRV-placebo, according to Student’s *t*-test. (**C**). The relative ATP content in comparison to cell density (OD_595nm_) for each sample. *n* = 3. * *p* < 0.05 for SRV-CBG versus SRV-placebo, according to Student’s *t*-test.

**Figure 3:**
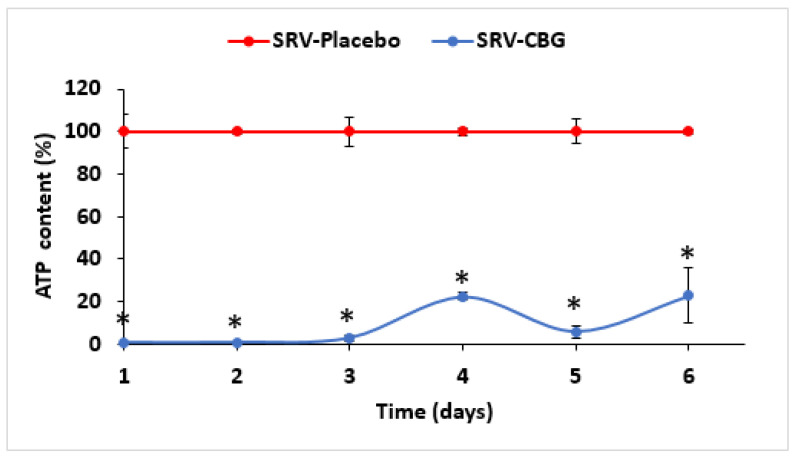
Antibacterial effects of SRV-CBG-coated mesh on planktonic growth of a clinical MDRSA CI-M isolate. SRV-CBG- and SRV-placebo-coated meshes were incubated daily with fresh MDRSA CI-M cultures for 6 days. The relative ATP content in MDRSA CI-M planktonic bacteria exposed to SRV-CBG-coated meshes was reduced by 78–99% compared to ATP contents in planktonic MDRSA CI-M bacteria exposed to SRV-placebo-coated meshes. The kinetic study was conducted for 6 days. *n* = 3. * *p* < 0.05 for SRV-CBG versus SRV-placebo, according to Student’s *t*-test.

To test the antibacterial effect of CBG-coated meshes against *S. aureus*, the coated meshes were incubated daily with fresh *S. aureus* cultures for 24 h for a period of 20 days, and the optical densities (ODs) of the planktonic-growing bacteria were measured. The SRV-CBG-coated meshes prevented *S. aureus* growth for 9 days (86 ± 4%) when compared with the SRV-placebo-coated meshes (Figure 2A; *p* < 0.05 when comparing SRV-CBG with SRV-placebo). After day nine, the OD of the *S. aureus* grown in the presence of the SRV-CBG-coated meshes approached that of the bacteria grown in the presence of SRV-placebo-coated meshes with no significant differences (Figure 2A). However, when the ATP content of the bacteria was analyzed using the BacTiter-Glo microbial cell viability assay, it was found to be lower in bacteria exposed to SRV-CBG than in those exposed to SRV-placebo, even during days 10–17 (Figure 2B; *p* < 0.05), suggesting that the amount of CBG released was sufficient to decrease the metabolic activity of the bacteria. The ATP content of *S. aureus* exposed to SRV-CBG was significantly reduced by 98.8 ± 1.3% in the first 9 days, whereas the ATP content on days 10–17 was still only 12–30% of that observed in bacteria exposed to SRV-placebo (Figure 2B). A similar antibacterial effect was observed toward a multiple-drug resistant *S. aureus* (MDRSA) clinical isolate (CI-M) (Figure 3), where the ATP content of planktonic MDRSA CI-M exposed to the SRV-CBG-coated mesh was significantly reduced by 78–99% in the first 6 days in comparison to MDRSA CI-M bacteria exposed to the SRV-placebo-coated mesh (Figure 3; *p* < 0.05). When the ATP content of each sample was divided by its own OD to compare the ATP content to cell density, the relative reduction in the ATP content of the SRV-CBG-exposed bacteria remained high in comparison to SRV-placebo-exposed bacteria (Figure 2C), indicating a net reduction in ATP content per cell.

### 2.3. SRV-CBG-Coated VICRYL Meshes Prevented Biofilm Formation in Its Surroundings

**Figure 4:**
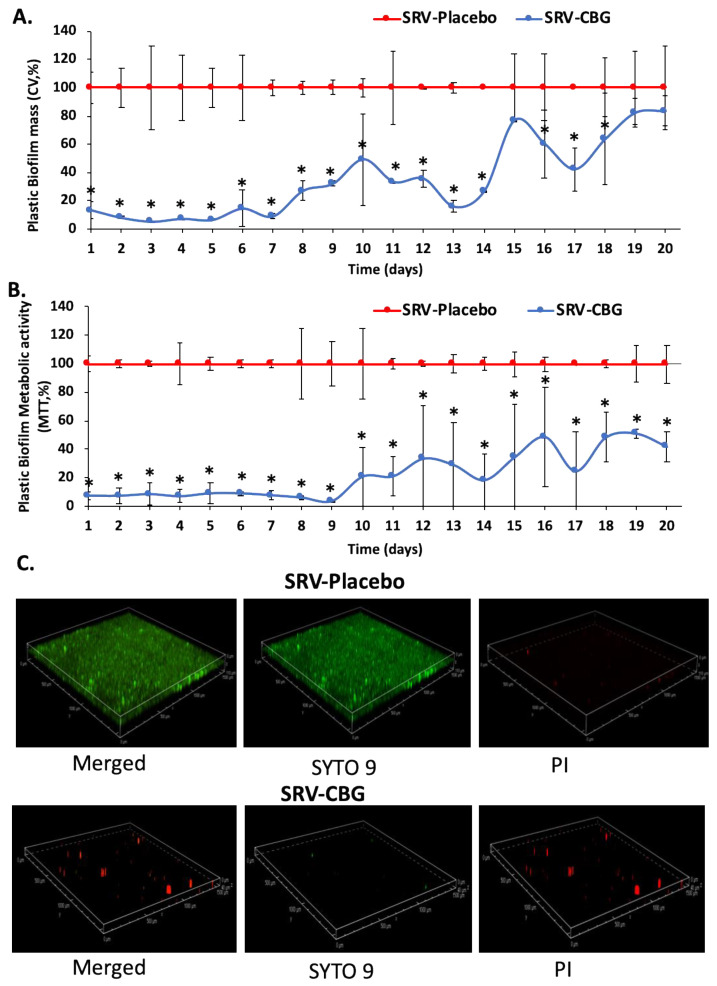
(**A**). Biofilm mass of *S. aureus* formed on the plastic well after daily exposure to SRV-CBG-coated mesh or SRV-placebo-coated mesh, as determined by crystal violet staining. *n* = 3. * *p* < 0.05 for SRV-CBG versus SRV-placebo, according to Student’s *t*-test. (**B**). Biofilm metabolic activity of *S. aureus* exposed to SRV-CBG-coated mesh compared with biofilm metabolic activity of *S. aureus* exposed to SRV-placebo-coated mesh, as determined by using MTT assay. *n* = 3. * *p* < 0.05 for SRV-CBG versus SRV-placebo, according to Student’s *t*-test. (**C**). SYTO 9/PI staining of *S. aureus* biofilm formed on the plastic well after being exposed to SRV-placebo (upper row) and SRV-CBG-coated mesh (lower row) segments on day 5 of incubation.

**Figure 5:**
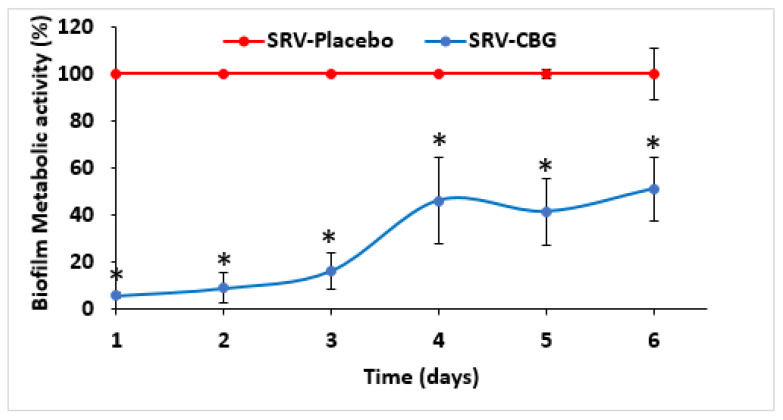
Antibiofilm effects of SRV-CBG-coated meshes on a clinical MDRSA CI-M isolate. SRV-CBG- and SRV-placebo-coated meshes were incubated daily with fresh MDRSA CI-M cultures for 6 days. The metabolic activity of MDRSA CI-M biofilms formed following incubation with SRV-CBG-coated mesh was reduced by 50–95% compared with the metabolic activity of MDRSA CI-M biofilms formed following incubation with SRV-placebo-coated mesh, as determined by using MTT assay. *n* = 3. * *p* < 0.05 for SRV-CBG versus SRV-placebo, according to Student’s *t*-test.

It was also important to investigate the antibiofilm effect of the SRV-CBG-coated mesh against *S. aureus*. To this end, the biofilm formed on the bottom of each well was stained daily with crystal violet (CV) for 20 days. The SRV-CBG-coated mesh had a strong antibiofilm effect for 7 days, with an 85–95% reduction in biofilm biomass compared with biofilms formed in the presence of the SRV-placebo-coated mesh (Figure 4A; *p* < 0.05). At days 7–14, there was still a 50–85% reduction in biofilm mass with some fluctuations that could be explained by uneven CBG release at these time points (Figure 4; *p* < 0.05). After that, the antibiofilm effect was only small (Figure 4A). There was also a strong reduction in the metabolic activity of biofilms formed in the presence of the SRV-CBG-coated mesh, which endured until day nine (Figure 4B; 92–98% reduction compared with SRV-placebo with a *p* < 0.05). Thereafter, there was only a partial inhibition of the metabolic activity up to day 20 (Figure 4B; 50–80% inhibition in comparison to SRV-placebo with a *p* < 0.05). An antibiofilm effect was also observed against an MDRSA clinical isolate (MDRSA CI-M) (Figure 5). The SRV-CBG-coated mesh had a strong antibiofilm effect for 6 days against MDRSA CI-M, with a 50–95% reduction in biofilm metabolic activity compared with biofilms formed in the presence of the SRV-placebo-coated mesh (Figure 5; *p* < 0.05).

**Figure 6:**
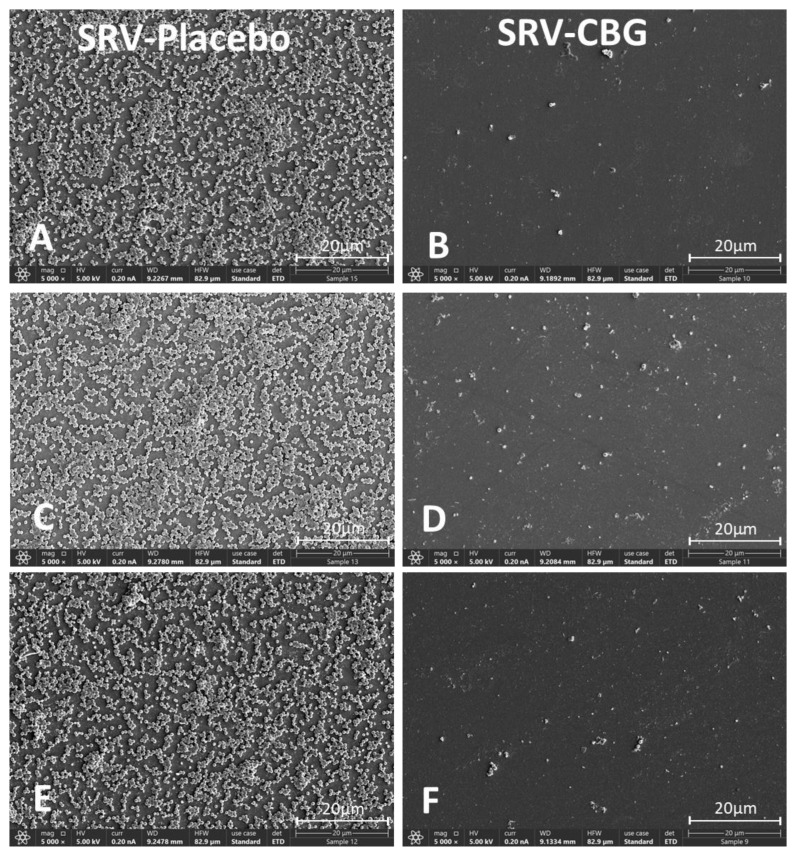
HR-SEM images of biofilms formed on the plastic well 24 h after the fifth exposure of SRV-placebo (**A**,**C**,**E**) or SRV-CBG-coated (**B**,**D**,**F**) meshes to *S. aureus*. A ×5000 magnification is shown. Images from three different samples of each group are shown.

To further demonstrate the antibiofilm effect of the SRV-CBG-coated mesh, the biofilms formed on the area adjacent to the mesh after the fifth exposure of the coated mesh to *S. aureus* were examined by using SDCM and HR-SEM. SDCM imaging was performed on biofilms formed on the plastic after live (SYTO 9)/dead (PI) staining (Figure 4C). The biofilms formed by *S. aureus* exposed to SRV-placebo appeared with strong SYTO 9 (green) fluorescence staining and only sporadic PI (red) fluorescence (Figure 4C, upper row), indicating the formation of biofilms of predominantly live bacteria. In contrast, *S. aureus* that was exposed to the SRV-CBG-coated mesh hardly formed biofilms, and only scattered green (SYTO 9) and red (PI) fluorescence could be detected, indicating the presence of only a few attached bacteria (Figure 4C, lower row). The HR-SEM imaging showed that the biofilms of *S. aureus* consisting of bacterial clusters formed on the plastic of samples exposed to the SRV-placebo-coated mesh (Figure 6A,C,E), whereas only a few scattered bacteria were present in the samples exposed to the SRV-CBG-coated mesh (Figure 6B,D,F). Overall, these data support the antibiofilm activity of the SRV-CBG coating against *S. aureus*.

### 2.4. SRV-CBG-Coated VICRYL Meshes Prevented the Adhesion of S. aureus to the Meshes

**Figure 7:**
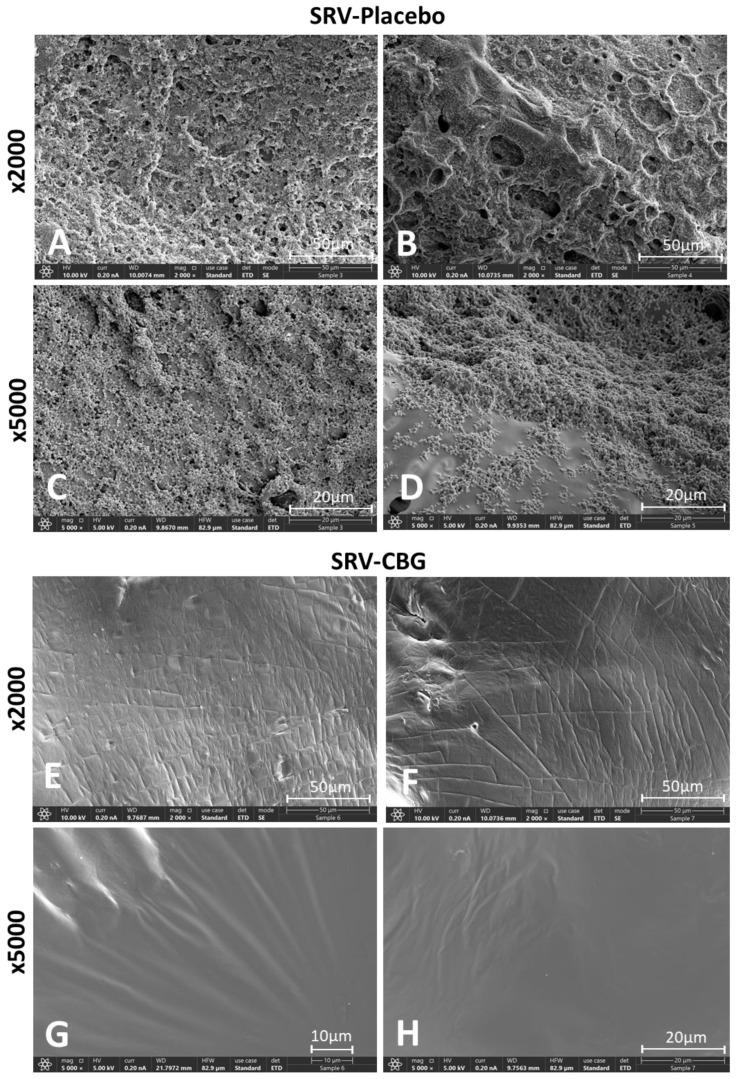
(**A**–**H**) HR-SEM images of SRV-placebo (**A**–**D**) and SRV-CBG-coated (**E**–**H**) mesh surfaces after 5 days of incubation with *S. aureus*. Magnifications of ×2000 (**A**,**B**,**E**,**F**) and ×5000 (**C**,**D**,**G**,**H**) are shown.

**Figure 8:**
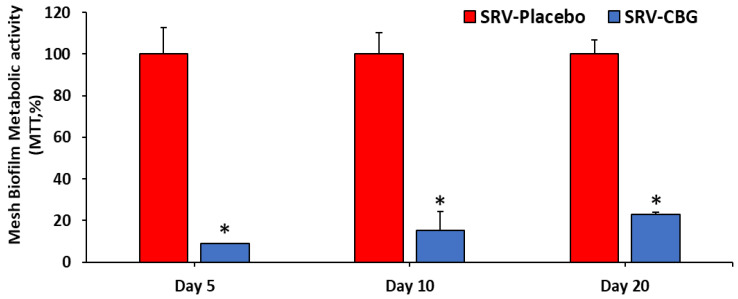
Metabolic activity of the biofilms formed on the coated meshes, as determined by using the MTT assay. The metabolic activity of SRV-placebo-coated mesh was set to 100%. *n* = 3. * *p* < 0.05 for SRV-CBG versus SRV-placebo, according to Student’s *t*-test.

When visualizing the *S. aureus* biofilms formed on the meshes themselves, the HR-SEM images showed a strong antibiofilm effect of the SRV-CBG-coated mesh compared with the SRV-placebo-coated mesh (Figure 7A–H). The SRV-placebo-coated meshes were covered by a biofilm of *S. aureus* appearing in clusters (Figure 7A–D), whereas only very few scattered *S. aureus* cells were visible on the SRV-CBG-coated mesh (Figure 7E–H). The prevention of biofilm formation on the SRV-CBG-coated mesh was also observed using the MTT metabolic assay (Figure 8). There was an 80–95% reduction in metabolic activity compared with SRV-placebo even after 20 days of exposure to *S. aureus*. Thus, our technique is effective in preventing biofilm formation not only in the surroundings, but also on the prosthetic material.

### 2.5. The SRV-CBG-coated Mesh was Not Cytotoxic to Vero Epithelial Cells

**Figure 9:**
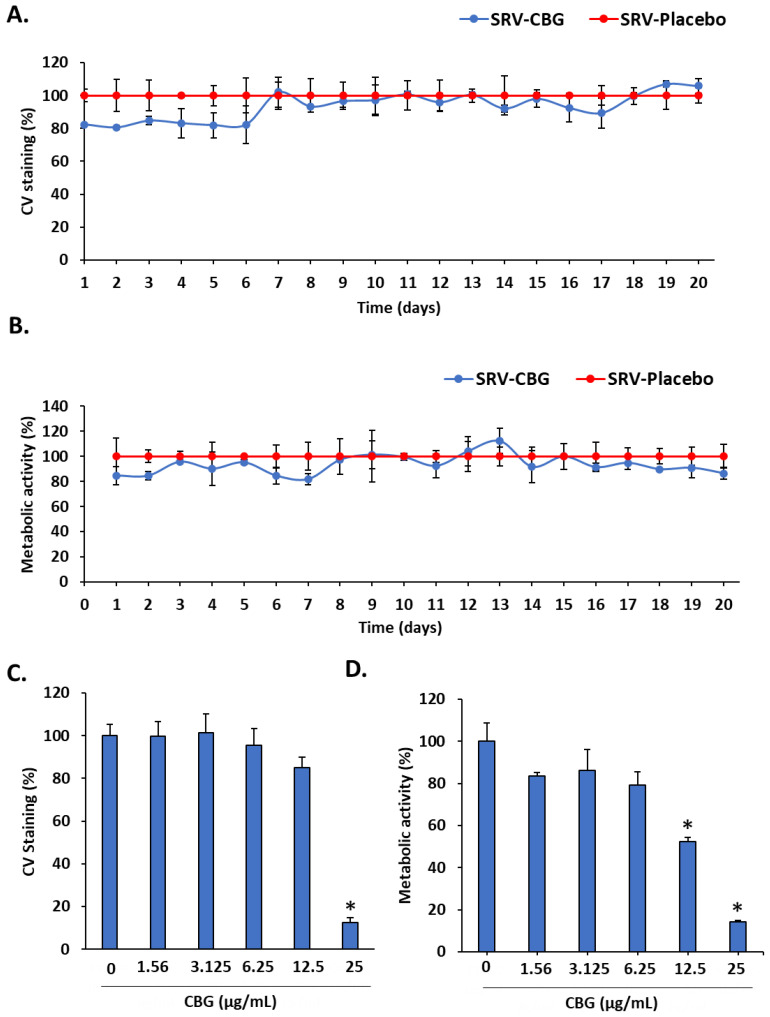
Biocompatibility assay on Vero epithelial cells. (**A**,**B**) Vero cell monolayer was exposed for 24 h to the daily collected DMEM-conditioned medium from SRV-CBG- and SRV-placebo-coated meshes for a period of 20 days. At the end of incubation, the cells were visualized under a light microscope and stained with CV for measuring the total cell mass (**A**) or exposed to MTT to measure the metabolic activity (**B**). *n* = 3. (**C**,**D**) Vero cell monolayer was incubated in the absence or presence of increasing concentrations of CBG (0–25 µg/mL) for 24 h and then either stained with CV for measuring their total cell mass (**C**) or MTT for measuring their metabolic activity (**D**). *n* = 3 * *p* < 0.05 in comparison to control (no CBG), according to Student’s *t*-test.

To test the biocompatibility of the SRV-CBG-coated mesh, the SRV-CBG or SRV-placebo-coated meshes were repeatedly incubated in DMEM for 24 h for a total of 20 days, and the conditioned medium was added to Vero cell monolayers in the presence of 10% FCS. Following a 24 h incubation period, the cultures were inspected visually by using light microscopy, the cell number was analyzed by CV staining, and their metabolic activity was determined by using the MTT assay. Light microscopy showed that there was no morphological difference between Vero cells treated with the conditioned medium from the SRV-placebo-coated mesh and SRV-CBG-coated mesh. CV staining showed a slight reduction in cell mass of approximately 20% in the SRV-CBG-exposed samples during the first six days, with no difference to SRV-placebo at later time points (Figure 9A). The metabolic activity of the cells exposed to the conditioned medium showed similar results (Figure 9B). Thus, the CBG concentrations released by the mesh were not toxic to Vero cells. CBG as a single agent showed an IC_50_ of 25 µg/mL on Vero cells (Figure 9C,D). Thus, the incorporation of CBG into the sustained-release varnish reduced its cytotoxicity towards epithelial cells while retaining its antibacterial and antibiofilm activities.

### 2.6. SRV-CBG-coated Mesh Reduces Cytokine Secretion by Macrophages

**Figure 10:**
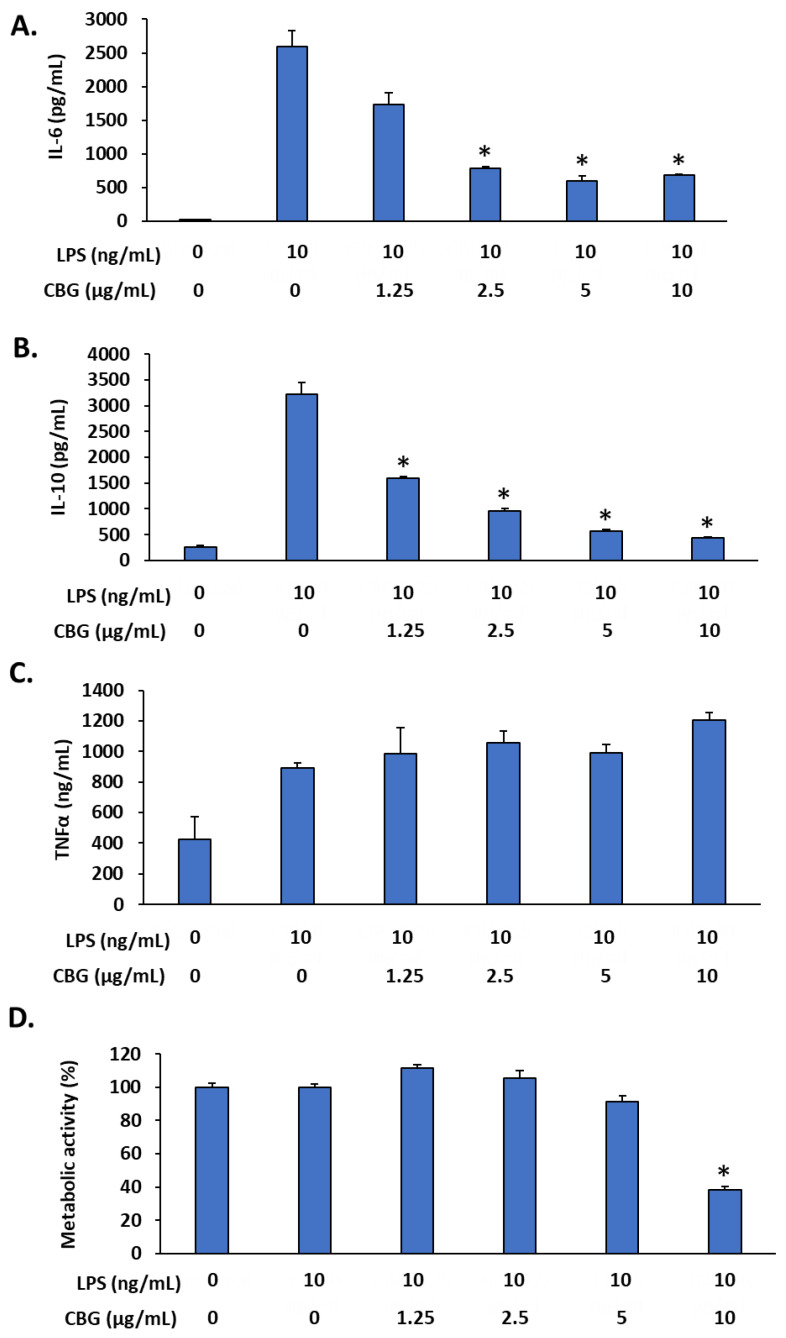
CBG inhibited LPS-induced IL-6 and IL-10 secretion but not TNFα secretion from RAW 264.7 macrophages. (**A**–**C**) RAW 264.7 macrophages were exposed to increasing concentrations of CBG in the presence of LPS (10 ng/mL) for 24 h, and the IL-6 (**A**), IL-10 (**B**), and TNFα (**C**) levels were analyzed by using respective ELISA kits. (**D**) The metabolic activity of macrophages after a 6 h incubation period with various concentrations of CBG and/or LPS. *n* = 3; * *p* < 0.05 in comparison to LPS-treated cells, according to Student’s *t*-test.

**Figure 11:**
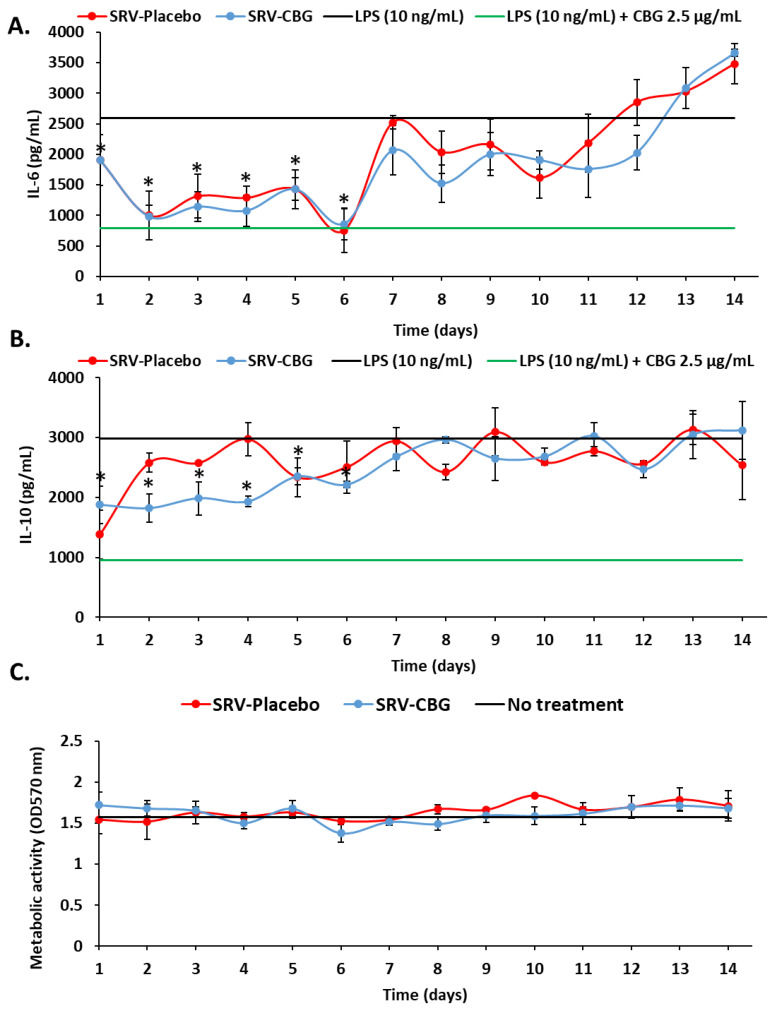
SRV-CBG-coated meshes inhibited LPS-induced IL-6 and IL-10 secretion from RAW 264.7 macrophages. (**A**,**B**) The levels of IL-6 (**A**) and IL-10 (**B**) secreted by RAW 264.7 macrophages after exposure to the conditioned medium collected from SRV-placebo and SRV-CBG for 14 days and 10 ng/mL LPS. The black and green lines represent the amount of IL-6 and IL-10 secreted by the same macrophages in the presence of LPS (10 ng/mL) (black line) or LPS (10 ng/mL) with CBG (2.5 µg/mL) (green line), respectively. *n* = 3. * *p* < 0.05 in comparison to LPS-treated cells, according to Student’s *t*-test. (**C**) Metabolic activity of the RAW 264.7 macrophages exposed to the conditioned medium of SRV-CBG and SRV-placebo for 14 days, as determined by using the MTT assay. The black line shows the MTT values of untreated macrophages. *n* = 3.

It was also important to investigate whether SRV-CBG retained the anti-inflammatory activity of CBG. We first examined whether CBG could prevent cytokine secretion using the RAW 264.7 macrophage cell line. We observed that CBG prevented LPS-induced IL-6 (Figure 10A) and IL-10 (Figure 10B) secretion in a dose-dependent manner, but not of TNFα (Figure 10C) secretion by the macrophages. CBG at 10 µg/mL caused a 60 ± 2% reduction in the metabolic activity of the macrophages (Figure 10D). We, therefore, analyzed the effect of the conditioned medium of SRV-CBG and SRV-placebo on LPS-induced IL-6 and IL-10 secretion (Figure 11). We observed that the conditioned medium of SRV-CBG significantly prevented the LPS-induced secretion of IL-6 and IL-10 (Figure 11A,B) for 6 days. Figure 11A shows that both the SRV-CBG and SRV-placebo inhibited IL-6 secretion significantly for up to 6 days (77 ± 7% inhibition), being almost similar to the IL-6 levels of the combined LPS (10 ng/mL) and CBG (2.5 µg/mL) treatment. Thereafter, the anti-inflammatory effect of both the SRV-placebo and SRV-CBG was gradually diminished until reaching the level of IL-6 induced by LPS (10 ng/mL) by day 14. The SRV-CBG varnish had a partial inhibitory effect on LPS-induced IL-10 secretion (26–36% inhibition) during the first 6 days with only a minor effect thereafter (Figure 11B). The SRV-placebo varnish had only a minor effect on the IL-10 secretion (14 ± 2% inhibition) during the entire tested period of 20 days (Figure 11B). This suggests that some components of the SRV have an anti-inflammatory effect. Noteworthily, neither the conditioned medium of SRV-CBG nor that of the SRV-placebo were cytotoxic to the RAW 264.7 macrophages, as determined by using the MTT assay (Figure 11C).

## 3. Discussion

Surgical mesh is used in various surgical areas to support the abdominal wall, where it contributes to tension-free tissue repairs and relieves perioperative pain. Its use shortens hospital stays and reduces recurrence rates. However, its application is also associated with some disadvantages, such as surgical site infections and inflammation [5]. Mesh infections are associated with high morbidity, hospital readmission, increased healthcare costs, repeat surgery, and impaired quality of life [43]. The occurrence of mesh infections is caused by multiple factors, including contamination during surgery, prolonged surgical time, and early wound complications [44]. Mesh biofilm infections are characterized by low-grade, chronic inflammation with minimal suppuration that can lead to mesh fibrosis and contraction, chronic pain, or even mechanical mesh failure [45].

One approach to combat mesh-associated infections is to develop surgical meshes with antimicrobial properties [13]. Strategies that have been taken into use include meshes coated with antimicrobial metals, antiseptics, and antibiotics [3,13,46]. Meshes coated with silver, chlorhexidine, or different combinations thereof are commercially available (DualMesh Plus^®^) [47,48]. Polypropylene (PP) meshes coated with nanocrystalline silver were bactericidal to *S. aureus*, as measured by the zone of inhibition after an 18 h incubation period [49]. Applying nanocrystalline-silver-coated PP meshes in an MRSA 25923 infection model in rats showed a 60% reduction in infection at the clinical surgical site compared with uncoated PP meshes [50]. PP meshes coated with a coladerm polymer with chlorhexidine were tested clinically in hernia patients and found to significantly reduce post-operative infections and complications [51]. The coating of PP meshes with a carboxymethylcellulose (CMC) gel loaded with chlorhexidine inhibited the growth of *S. aureus* (ATCC 25923), *Staphylococcus epidermidis* (ATCC 12228), and *Escherichia coli* (ATCC 25922) in a zone inhibition assay [52]. After 3 days, the entire amount of CHX is released from the coating [52]. The chlorhexidine CMC-coated PP reduced the viability of cultured fibroblasts [52], indicating a cytotoxic effect of chlorhexidine, making it a less attractive antimicrobial agent for coating meshes. Coating meshes with antibiotics such as gentamicin, vancomycin, and rifampicin are only efficient against antibiotic-sensitive strains, which is a significant limitation [53,54,55].

CBG is marketed as a dietary supplement [37], and in vivo studies have been performed in rodents showing that CBG is well tolerated and exerts anti-inflammatory and neuroprotective activities [36,37,56,57,58]. Because CBG also has antimicrobial activities, especially against Gram-positive bacteria including *S. aureus* [29,30,40,41], its efficacy was investigated when incorporated into a slow-release varnish (SRV) applied on a biodegradable Vicryl mesh.

Here, we show that the CBG-containing sustained-release varnish has a dual action, where it prevents the growth and biofilm formation of *S. aureus* and inhibits cytokine secretion (IL-6 and IL-10) from LPS-activated macrophages. The release of CBG from the coated meshes was efficient over a long period of time, and hence, the SRV-CBG is expected to provide prolonged protection. The reason why we chose CBG as the active compound in our study is that CBG has been shown to have both antibacterial and anti-inflammatory properties [29,37,40,41,59,60]. It is important to note that any mesh type can be used for our technology, but we focused here on VICRYL^®^ (polyglactin 910) mesh as a model because it is feasible, cost-effective, and clinically used in contaminated situations. Several devices with SRV technologies have already been developed and commercialized in various medical fields [26,61,62]. To the best of our knowledge, this is the first in vitro study of applying CBG in an SRV technology to coat surgical meshes for the purpose of preventing bacterial biofilm formation and inflammation at surgical sites. Because this drug delivery system is a pharmaceutical platform, different drugs can also be incorporated into the SRV and tested for their release kinetics and biological efficacy.

The first objective of our study was to investigate whether the varnish would be retained on our mesh and for how long it would provide an antibacterial effect. The SRV-CBG-coated mesh showed a strong antibacterial effect against *S. aureus* for 9 days. Thereafter, there was only partial inhibition of bacterial growth, which might be due to fluctuation in the amount of CBD released, leading to a concentration around the MIC value. By day 16, the amount of CBG released was no longer sufficient to prevent bacterial growth. However, still, up to day 17, the ATP content of the bacteria exposed to SRV-CBG-coated meshes was significantly reduced, which is consistent with the documented anti-metabolic effect of CBG [40]. Although bacterial growth began to increase after day 10, the ATP levels remained at 30% until day 17, which was shown to be due to the reduction in the net ATP content per cell rather than the reduced number of bacteria.

Because bacterial adherence to tissue, mesh, and the subsequent biofilm formation is one of the main causes of the poor management of staphylococcal infections [14], it was important to investigate the biofilm formation on the SRV-CBG-coated mesh as well as in its environment after repeated exposure to planktonic-growing *S. aureus*. The biofilms were quantified by using different methods, including CV biomass staining, MTT metabolic assay, HR-SEM, and SDCM imaging. It should be noted that each assay monitors different parameters. While CV stains the bacterial biofilm, MTT measures the metabolic activity of the bacteria, HR-SEM visualizes the biofilm, and SDCM can distinguish between live and dead bacteria following SYTO 9/PI staining. Even after five exposures of the coated meshes to *S. aureus*, there were almost no bacteria adhered to the SRV-CBG-coated mesh segments, which appeared smooth with only scattered single cells visible. In contrast, the SRV-placebo-coated meshes exposed to *S. aureus* under the same conditions were heavily covered by *S. aureus* biofilms. The metabolic activity of the biofilm formed on the SRV-CBG-coated mesh was significantly reduced to 20 ± 1% even at day 20, indicating that the SRV-CBG coating prevents bacterial adherence to the mesh.

We further observed that the biofilm biomass in the environment was significantly decreased when exposed to the SRV-CBG-coated mesh during the first week. Although *S. aureus* bacteria showed their full growth and biofilm formation ability after day 15, they did not regain their full metabolic activity. This might be related to the anti-metabolic activity of CBG [40,59]. Because surgical wound infections usually occur in the first days after surgery, we chose day five to visualize the bacterial biofilm by using HR-SEM and SDCM. HR-SEM and SDCM confirmed the reduction in the biofilm mass in the surroundings around the SRV-CBG-coated mesh. The same trend was seen with both imaging techniques, where scattered single cells were visible in the vicinity of the SRV-CBG-coated meshes. Some scattered PI staining could be seen in SDCM, which could be some dead bacteria adhered to the plastic well surface. Altogether, these data show the efficiency of the SRV-CBG-coated meshes in preventing both bacterial growth and biofilm formation not only on the meshes but also in their vicinity for a relatively long period. It is noteworthy to mention that similar antibacterial and antibiofilm effects of the SRV-CBG-coated meshes were observed against a clinical MDRSA isolate, indicating that this technology is also effective against drug-resistant *S. aureus* species.

The action mechanism of CBG is only partially understood. There is evidence that it targets the cytoplasmic membrane of Gram-positive bacteria, resulting in altered membrane potential, membrane permeability, and membrane fluidity [29,40]. The antibiofilm effect of CBG on *S. aureus* could be indirectly due to its antibacterial effect and directly due to its effects on metabolic pathways regulating biofilm formation, as shown for the Gram-positive bacteria *Streptococcus mutans* [59]. The closely related cannabidiol (CBD) was shown to exert anti-metabolic effects on *S. aureus* [63], and it is likely that this also holds true for CBG.

To investigate the anti-inflammatory effects of our SRV-CBG-coated meshes, we used the in vitro model of RAW264.7 macrophages stimulated with LPS. The SRV-CBG-coated meshes significantly reduced IL-6 and IL-10 secretion by the macrophages over a period of up to 6 days. Consistent with our results, it has been reported that CBG can reduce the secretion of IL-6 and IL-10 in peripheral blood mononuclear cell cultures [33]. Surprisingly, the SRV-placebo-coated meshes also possessed some anti-inflammatory properties. The immunosuppressive effect of SRV-placebo might be due to the presence of PEG 400, which has been shown to reduce the expression of inflammatory cytokines in LPS and zymosan models of sepsis [64]. Our results are also in line with a report of Cataldo Russomando et al. [65], where nasal stents coated with SRV-placebo exhibited anti-inflammatory properties. In fact, the presence of PEG 400 in the varnish might even be beneficial by increasing the anti-inflammatory effect of CBG. The inhibitory effect of SRV-CBG-coated mesh on IL-6 and IL-10 secretion suggests that this drug system may decrease inflammation at surgical sites.

An advantage of the SRV coating is that the drug is released in small portions over a longer period of time and the drug is delivered locally, thus reducing systemic side effects and improving safety [66]. The drug concentrations should be sufficient to exert the desired biological effects without harming the host. It was, therefore, important to investigate whether our SRV-CBG technology was not cytotoxic to normal epithelial cells. Indeed, using the well-accepted Vero epithelial cell model, which is considered the gold standard for drug testing according to the recommendations of ISO 10993-5 (2009) [67], we observed that the daily release of CBG from the coated mesh was below the cytotoxic concentration of the drug. This finding is important because we can achieve the desired biological therapy, such as preventing bacterial growth, biofilm formation, and suppressing the immune responses, while avoiding tissue damage. Further preclinical and in vivo toxicology studies are required to prove the applicability, biocompatibility, and safety of the SRV-CBG-coated mesh.

The objective of this study was to develop a sustained-release technology that can release enough CBG daily to prevent *S. aureus* growth and inhibit biofilm formation both in the surroundings and on the mesh for extended periods of time. In this in vitro model, the meshes were exposed daily to fresh *S. aureus* cultures to study whether the daily remaining CBG was still able to inhibit bacterial growth and biofilm formation, which was demonstrated. This in vitro model differs from in vivo conditions, where any bacterial infection occurring during surgery is expected to be eliminated by the SRV-CBG-coated mesh already during the first day post-operation. One of the limitations of this study is that it was conducted in vitro using only two bacterial strains (an MSSA and an MDRSA clinical isolate) and one inflammatory model (macrophages). Having proven the pharmaceutical concept of our platform, further studies should focus on defining the bacterial spectrum targeted by CBG, its biosafety, and its in vivo effects.

In conclusion, we demonstrated the efficacy of an SRV technique in vitro in which CBG is released in a sustained-released manner, providing protection against the bacterial growth and biofilm formation of *S. aureus*, a major pathogen involved in mesh-related infections. Moreover, we presented data demonstrating the anti-inflammatory properties of this device. This research provides the basis for further preclinical in vivo studies and may have implications for the future treatment of surgical-mesh-associated infections.

## 4. Materials and Methods

### 4.1. Materials

The synthetic biodegradable mesh used for this study was VICRYL mesh (polyglactin 910), which was obtained from Ethicon Inc. (Raritan, NJ, USA). Cannabigerol (CBG) (95% pure hemp isolate) was purchased from NC Labs (Prague, Czech Republic). 3-(4,5-Dimethyl-2-thiazolyl)-2,5-diphenyl-2H-tetrazolium bromide (MTT) and polyethylene glycol 400 (PEG 400) were obtained from Sigma Aldrich (St. Louis, MO, USA). Hydroxypropyl cellulose (Klucel™ EF-grade) was obtained from Ashland Specialty Ingredients (Wilmington, DE, USA), and aminomethacrylate copolymer (Eudragit™ E100) was obtained from Hercules Inc. (Wilmington, DE, USA). Absolute ethanol (HPLC-grade) was obtained from J. T. Baker (Gliwice, Poland).

### 4.2. Formulation of the Sustained-Release Varnish (SRV)

The sustained-release varnish of CBG (SRV-CBG) was prepared by mixing 35.8 mg of PEG 400, 179 mg of Eudragit™ E100, and 107 mg of Klucel™ EF in 5 mL of absolute ethanol to which 214.8 mg CBG was added. Placebo varnish, which was prepared in a similar manner without CBG, was used as the control [68].

### 4.3. Coating the Mesh with the Varnish

Here, 1 × 1 cm segments of VICRYL meshes (polyglactin 910) (Ethicon, NJ, USA) were disinfected in 70% ethanol and dried at room temperature (RT). The disinfected meshes were then triple-coated with SRV-CBG or SRV-placebo, which was performed by immersing the meshes in the SRV for 30 s, and then air-dried until a film was formed on the segments. For each coating, the mesh was allowed to dry for at least 40 min at room temperature. The process was repeated twice, resulting in 60–70 mg of dry SRV film per mesh piece. The coated meshes were allowed to dry completely at room temperature overnight before use [27].

### 4.4. Staphylococcus aureus Growth Conditions

A frozen stock of *S. aureus* ATCC 25923, MRSA ATCC 33592, MRSA ATCC 43300, Newman MRSA, and a clinical multidrug-resistant *S. aureus* (MDRSA CI-M) [42] were inoculated into tryptic soy broth (TSB) (Himedia, PA, USA) at a ratio of 1:100 and incubated overnight at 37 °C until an OD_600nm_ of 1.8–2.0 was reached. The overnight bacterial culture was diluted in fresh TSB medium to an OD_600nm_ of 0.1 and used for the biological assays described below.

### 4.5. Determination of Minimum Inhibitory Concentration (MIC) of CBG

The various *S. aureus* strains were diluted to an initial OD_600nm_ of 0.1 in TSB containing 1% D-glucose and exposed to increasing concentrations of CBG (0–10 µg/mL) in a volume of 200 µL in a tissue-grade flat-bottom 96-well microplate (Corning, Glendale, AZ, USA). After a 24 h incubation period at 37 °C, the OD at 600 nm was measured in a Tecan Infinite M200 PRO plate reader (Tecan Trading AG, Männedorf, Switzerland) [42]. The MIC was determined as the lowest concentration of CBG that did not cause any visible growth. Quantification of the biofilms formed at the surface was determined by crystal violet staining and MTT metabolic activity as described [42]. The MBIC was determined as the lowest concentration that prevented biofilm formation after a 24 h incubation period.

### 4.6. S. aureus Planktonic Growth and Biofilm Formation in the Presence of SRV-Coated Mesh

An amount of 1.5 mL of the diluted *S. aureus* ATCC 25923 culture in TSB was added to each mesh coated with either SRV-CBG or SRV-placebo in a flat-bottom 12-well microplate (Corning, Incorporated, Kennebunk, ME, USA) and then incubated at 37 °C for 24 h. On the following day, the OD_595nm_ of the supernatant was measured using a Tecan M200 infinite microplate reader (Tecan Trading AG, Männedorf, Switzerland) to measure the planktonic bacterial growth. TSB medium incubated with the mesh without bacteria served as a blank. The meshes were transferred daily to fresh *S. aureus* cultures in TSB medium for 20 days. The biofilms formed at the bottom of the wells were washed twice with phosphate-buffered saline (PBS) (Sartorius, Beit HaEmek, Israel) and analyzed using the different techniques described below.

### 4.7. Measuring the Metabolic Activity of Planktonic Bacteria

Quantification of the ATP content in planktonic bacteria was performed using the BacTiter-Glo microbial cell viability assay (Promega, WI, USA). An amount of 100 µL of the reagent was mixed with an equal volume of each sample in 96-well flat-bottom plates (Greiner Bio-One, μClear white clear-bottom plates, Frickenhausen, Germany), and after shaking for 5 min on an orbital shaker, the luminescence was recorded using an M200 Infinite Tecan plate reader (Tecan Trading AG, Switzerland) [40]. The level of ATP of SRV-CBG samples was calculated in comparison to the ATP level of SRV-placebo samples each day using the following equation: (SRV-CBG luminescence/SRV-placebo luminescence) × 100. The relative ATP content in comparison to cell density (OD_595nm_) was calculated using the following formula: (SRV-CBG luminescence/SRV-CBG OD_595nm_) × 100 and (SRV-placebo luminescence/SRV-placebo OD_595nm_) × 100.

### 4.8. Determination of S. aureus Biofilm Biomass by Crystal Violet (CV) Staining

The washed *S. aureus* biofilms were stained for 15 min with 500 µL of 0.1% crystal violet (CV) prepared from 0.4% Gram’s crystal violet solution (Merck, EMD Millipore Corporation, Billerica, MA, USA) diluted in double-distilled water (DDW) [59]. At the end of incubation, the wells were washed twice with DDW. An amount of 1 mL of 33% acetic acid was added to each well to extract the CV stain. The amount of biofilm biomass was quantified by measuring the absorbance of the CV stain at 595 nm using an M200 Infinite Tecan plate reader (Tecan Trading AG, Switzerland). The percentage of the biofilm biomass was quantified using the following equation: (SRV-CBG OD_595nm_/ SRV-placebo OD_595nm_) × 100.

### 4.9. Determination of S. aureus Biofilm Metabolic Activity Using Tetrazolium Reduction Assay

The metabolic activity of the biofilms was measured by incubating the biofilms formed on the wells or on the SRV-CBG- or SRV-placebo-coated meshes with 500 μL of a 0.5 mg/mL MTT solution in PBS for 2 h at 37 °C. The tetrazolium precipitate was extracted with 1 mL of dimethylsulfoxide (DMSO) (Bio-Lab Ltd., Jerusalem, Israel), and absorbance at 570 nm was measured using a Tecan M200 microplate reader (Tecan Trading AG, Switzerland) [69]. The percentage of metabolically active bacteria was quantified using the following equation: (SRV-CBG OD_570nm_/ SRV-placebo OD_570nm_) × 100.

### 4.10. High-Resolution Scanning Electron Microscopy (HR-SEM)

SRV-CBG/SRV-placebo-coated meshes and sterile glass pieces were inserted in flat-bottom 12-well microplates (Corning, Incorporated, Kennebunk, ME, USA) at day 4 of co-cultivation with *S. aureus*, and biofilms of *S. aureus* were allowed to form for 24 h. On the following day (day 5 of co-cultivation), the glass pieces or the CBG/placebo-coated meshes were rinsed with DDW and fixed in 4% glutaraldehyde (Electron Microscopy Sciences, Hatfield, PA, USA) in DDW for 40 min. After fixation, the pieces were washed again with DDW and allowed to dry at room temperature. The samples were then coated with iridium and visualized by using a high-resolution scanning electron microscope (Apreo 2 S, ThermoScientific, Waltham, MA, USA) [70].

### 4.11. Spinning Disk Confocal Microscopy (SDCM)

This assay was performed similarly to the method described in [71]. In brief, the *S. aureus* biofilms formed on day 5 at the bottom of a flat-bottom 12-well microplate (Corning, Incorporated, Kennebunk, ME, USA) were washed twice with PBS and stained with 3.3 µM SYTO 9 (Invitrogen, Life Technologies, Carlsbad, CA, USA) and 2 µg/mL propidium iodide (PI; Sigma, St. Louis, MO, USA) and kept in the dark at RT for 20 min. Following the incubation, the stained biofilms were washed with PBS and fixed in 4% paraformaldehyde for 40 min. After fixation, the biofilms were washed again with PBS and mounted with 1 mL of 50% glycerol in DDW. The stained biofilms were visualized under a Nikon Spinning Disk confocal microscope (SDCM) (Nikon Corporation, Tokyo, Japan) connected to Yokogawa W1 Spinning Disk (Yokogawa Electric Corporation, Tokyo, Japan). Imaris software (Imaris version 9.8.0., Oxford Instruments, 2021, Abingdon, UK) were used to construct three-dimensional (3D) image of the biofilms. More than three random fields from each sample were analyzed.

### 4.12. Cytotoxicity Assay on Vero Epithelial Cells

For the biocompatibility assay, we used normal Vero kidney epithelial cells (ATCC CCL-81), which were kindly provided by Dr. Alexander Rouvinski, the Hebrew University of Jerusalem, Israel. The 4 × 10^5^ Vero cells were seeded per well into 96-well flat-bottom tissue cultures (Corning) in 200 µL DMEM (Sigma) supplemented with 10% heat-inactivated fetal calf serum (Sigma), 2 mM L-glutamine, 1 mM sodium pyruvate, 100 U/mL penicillin, and 0.1 mg/mL streptomycin (Biological Industries, Beth HaEmek, Israel) and incubated at 37 °C with 5% CO_2_. On the following day, when a monolayer was formed, the medium was removed and exchanged with 200 µL of the daily collected medium from the meshes and further incubated for another 24 h. In parallel, the cells were incubated with various concentrations of CBG. At the end of incubation, the cells were stained with either 200 µL 0.1% CV at room temperature for 20 min or 0.5 mg/mL MTT solution in PBS for 45 min at 37 °C. The cells were then washed twice with PBS, and 200 µL of either 33% acetic acid or DMSO was added to each well to extract the CV stain or the formazan, respectively. The absorbance at 595 nm (for CV) and 570 nm (for MTT) was measured using an M200 infinite Tecan plate reader [72].

### 4.13. Cultivation of Mouse RAW 264.7 Macrophage Cell Line and the In Vitro Inflammation Model

The mouse RAW 264.7 macrophage cell line (ATCC^®^ TIB-7™) was kindly provided by Prof. Gabriel Nussbaum at the Faculty of Dental Medicine, The Hebrew University of Jerusalem, Israel. The macrophages were seeded at 4 × 10^5^ cells per well in 96-well flat-bottom tissue cultures (Corning) in 200 µL DMEM (Sigma) supplemented with 10% heat-inactivated fetal calf serum (Sigma), 2 mM L-glutamine, 1 mM sodium pyruvate, 100 U/mL penicillin, and 0.1 mg/mL streptomycin (Biological Industries, Beth HaEmek, Israel) and incubated at 37 °C with 5% CO_2_. On the following day, the medium was removed and exchanged with 100 µL of the daily collected medium from the meshes or media supplemented with different concentrations of CBG (0–5 µg/mL). After a 30 min incubation period at 37 °C, 100 µL of DMEM supplemented with 10% FCS and 20 ng/mL lipopolysaccharide (LPS; Sigma) was added to each well. Macrophages supplemented with 10% FCS without LPS served as control. The plate was then further incubated at 37 °C for another 24 h. At the end of incubation, the plate was centrifuged at 1500 rpm for 5 min, and the supernatants were collected for determining the cytokine content (TNFα, IL-6, IL-10) using the ELISA technique described below.

### 4.14. Determination of Cytokine Content by Enzyme-Linked Immunosorbent Assay (ELISA) 

Determination of the cytokine content (IL-10, IL-6, TNFα) in the supernatant of macrophages was performed according to the manufacturer’s instruction of ABTS ELISA developmental kits of Peprotech (Cranbury, NJ, USA; Cat# 900-M50 for IL-6, Cat# 900-M53 for IL-10; and Cat# 900-M54 for TNFα). The detection ranges were 63–4000 pg/mL for mouse IL-6; 39–5000 pg/mL for mouse IL-10; and 15–1000 pg/mL for TNFα. The cytokine levels were calculated against a standard curve made from the respective cytokines provided by the kit. The day before the ELISA, the captured antibody was diluted in PBS to a recommended concentration, and 100 µL was added to each well of an ELISA Maxisorb Nunc-immune plate (Thermo Scientific, Roskilde, Denmark) and left on a shaker at RT overnight. On the following day, the wells were washed four times with PBS supplemented with 0.05% Tween 20 (Bio-Rad, Hercules, CA, USA) and blocked with 300 µL of PBS containing 1% bovine serum albumin (BSA; VWR Chemicals, Fountain Pkwy Solon, OH, USA) for 1 h, followed by another four washes with PBS containing 0.05% Tween 20 (Bio-Rad, CA, USA). Then, 100 µL of two-fold dilutions of the cytokines or 50 µL of samples plus 50 µL diluent (PBS containing 0.05% Tween 20 and 0.1% BSA) were added to each well and incubated for 2 h at room temperature. For TNFα samples, a 1:100 dilution ratio of the samples was used. This was followed by four washes with PBS containing 0.05% and the addition of the detection antibody diluted in diluent to the recommended concentration and incubated for another 2 h at room temperature. The wells were again washed with PBS containing 0.05% Tween 20 and incubated with recommended concentrations of avidin-HRP conjugate (ABTS kit) for 30 min. Finally, the wells were washed again four times with PBS containing 0.05% Tween 20, and 100 µL of ABTS substrate (Cranbury, NJ, USA) was added to each well. The absorbance of the green color was measured at 405 nm using a Tecan M200 Infinite plate reader.

### 4.15. Statistical Analysis

Three independent experiments were performed for each assay. Within each experiment, triplicates were conducted for each sample. Student’s *t*-test was performed for statistical analysis of the data. A *p*-value of less than 0.05 was considered significant.

## Figures and Tables

**Table 1:** The susceptibility of MSSA and MRSA strains to CBG *.

*S. aureus* Strain	MIC (µg/mL)	MBIC (µg/mL)
MSSA ATCC 25923	2	2
MRSA ATCC 33592	2	2
MRSA ATCC 43300	2.5	2.5
Newman MRSA	2.5	2.5
MDRSA CI-M	2.5	2.5

* MSSA—methicillin-sensitive *S. aureus*; MRSA—methicillin-resistant *S. aureus*; MDRSA CI-M—a clinical isolate of a multidrug-resistant *S. aureus* (MDRSA), which is resistant to methicillin, gentamicin, norfloxacin, and erythromycin [42]. MIC—minimum inhibitory concentration, which is the lowest concentration resulting in non-visual growth after a 24 h incubation period. MBIC—minimum biofilm inhibitory concentration, which is the lowest concentration preventing biofilm formation after a 24 h incubation period.

## Data Availability

Raw data for the figures are available upon reasonable request from the corresponding author.
